# Supplementary material for: OpenMM 7: Rapid development of high performance algorithms for molecular dynamics
Source: PLoS Comput Biol. 2017 Jul 26;13(7):e1005659. doi: 10.1371/journal.pcbi.1005659 (PMC5549999; doi:10.1371/journal.pcbi.1005659)
Supplement: S1 Source Code — (ZIP) [file pcbi.1005659.s002.zip › OpenMM-7.0.1-Source/docs/api-c++/generated/OpenMM.CustomNonbondedForce.html]

CustomNonbondedForce — OpenMM @OPENMM\_MAJOR\_VERSION@.@OPENMM\_MINOR\_VERSION@ documentation


# CustomNonbondedForce¶

*class* `OpenMM::``CustomNonbondedForce`¶
:   This class implements nonbonded interactions between particles. Unlike `NonbondedForce`, the functional form of the interaction is completely customizable, and may involve arbitrary algebraic expressions and tabulated functions. It may depend on the distance between particles, as well as on arbitrary global and per-particle parameters. It also optionally supports periodic boundary conditions and cutoffs for long range interactions.

    To use this class, create a `CustomNonbondedForce` object, passing an algebraic expression to the constructor that defines the interaction energy between each pair of particles. The expression may depend on r, the distance between the particles, as well as on any parameters you choose. Then call `addPerParticleParameter()` to define per-particle parameters, and `addGlobalParameter()` to define global parameters. The values of per-particle parameters are specified as part of the system definition, while values of global parameters may be modified during a simulation by calling `Context::setParameter()`.

    Next, call `addParticle()` once for each particle in the `System` to set the values of its per-particle parameters. The number of particles for which you set parameters must be exactly equal to the number of particles in the `System`, or else an exception will be thrown when you try to create a `Context`. After a particle has been added, you can modify its parameters by calling `setParticleParameters()`. This will have no effect on Contexts that already exist unless you call `updateParametersInContext()`.
    `CustomNonbondedForce` also lets you specify “exclusions”, particular pairs of particles whose interactions should be omitted from force and energy calculations. This is most often used for particles that are bonded to each other.

    As an example, the following code creates a `CustomNonbondedForce` that implements a 12-6 Lennard-Jones potential:

    ```
    CustomNonbondedForce* force = new CustomNonbondedForce("4*epsilon*((sigma/r)^12-(sigma/r)^6); sigma=0.5*(sigma1+sigma2); epsilon=sqrt(epsilon1*epsilon2)");
    ```

    This force depends on two parameters: sigma and epsilon. The following code defines these as per-particle parameters:

    ```
    force->addPerParticleParameter("sigma");
    force->addPerParticleParameter("epsilon");
    ```

    The expression
    `CustomNonbondedForce` can operate in two modes. By default, it computes the interaction of every particle in the `System` with every other particle. Alternatively, you can restrict it to only a subset of particle pairs. To do this, specify one or more “interaction groups”. An interaction group consists of two sets of particles that should interact with each other. Every particle in the first set interacts with every particle in the second set. For example, you might use this feature to compute a solute-solvent interaction energy, while omitting all interactions between two solute atoms or two solvent atoms.

    To create an interaction group, call `addInteractionGroup()`. You may add as many interaction groups as you want. Be aware of the following:

    > - Exclusions are still taken into account, so the interactions between excluded pairs are omitted.
    > - Likewise, a particle will never interact with itself, even if it appears in both sets of an interaction group.
    > - If a particle pair appears in two different interaction groups, its interaction will be computed twice. This is sometimes useful, but be aware of it so you do not accidentally create unwanted duplicate interactions.
    > - If you do not add any interaction groups to a `CustomNonbondedForce`, it operates in the default mode where every particle interacts with every other particle.

    When using a cutoff, by default the interaction is sharply truncated at the cutoff distance. Optionally you can instead use a switching function to make the interaction smoothly go to zero over a finite distance range. To enable this, call `setUseSwitchingFunction()`. You must also call `setSwitchingDistance()` to specify the distance at which the interaction should begin to decrease. The switching distance must be less than the cutoff distance. Of course, you could also incorporate the switching function directly into your energy expression, but there are several advantages to keeping it separate. It makes your energy expression simpler to write and understand. It allows you to use the same energy expression with or without a cutoff. Also, when using a long range correction (see below), separating out the switching function allows the correction to be calculated more accurately.

    Another optional feature of this class is to add a contribution to the energy which approximates the effect of all interactions beyond the cutoff in a periodic system. When running a simulation at constant pressure, this can improve the quality of the result. Call `setUseLongRangeCorrection()` to enable it.

    Computing the long range correction takes negligible work in each time step, but it does require an expensive precomputation at the start of the simulation. Furthermore, that precomputation must be repeated every time a global parameter changes (or when you modify per-particle parameters by calling `updateParametersInContext()`). This means that if parameters change frequently, the long range correction can be very slow. For this reason, it is disabled by default.

    Expressions may involve the operators + (add), - (subtract), \* (multiply), / (divide), and ^ (power), and the following functions: sqrt, exp, log, sin, cos, sec, csc, tan, cot, asin, acos, atan, sinh, cosh, tanh, erf, erfc, min, max, abs, floor, ceil, step, delta, select. All trigonometric functions are defined in radians, and log is the natural logarithm. step(x) = 0 if x is less than 0, 1 otherwise. delta(x) = 1 if x is 0, 0 otherwise. select(x,y,z) = z if x = 0, y otherwise. The names of per-particle parameters have the suffix “1” or “2” appended to them to indicate the values for the two interacting particles. As seen in the above example, the expression may also involve intermediate quantities that are defined following the main expression, using ”;” as a separator.

    In addition, you can call `addTabulatedFunction()` to define a new function based on tabulated values. You specify the function by creating a `TabulatedFunction` object. That function can then appear in the expression.

    Methods

    |  |  |
    | --- | --- |
    | `CustomNonbondedForce` | Create a `CustomNonbondedForce`. |
    | `CustomNonbondedForce` |  |
    | `~CustomNonbondedForce` |  |
    | `getNumParticles` | Get the number of particles for which force field parameters have been defined. |
    | `getNumExclusions` | Get the number of particle pairs whose interactions should be excluded. |
    | `getNumPerParticleParameters` | Get the number of per-particle parameters that the interaction depends on. |
    | `getNumGlobalParameters` | Get the number of global parameters that the interaction depends on. |
    | `getNumTabulatedFunctions` | Get the number of tabulated functions that have been defined. |
    | `getNumFunctions` | Get the number of tabulated functions that have been defined. |
    | `getNumInteractionGroups` | Get the number of interaction groups that have been defined. |
    | `getEnergyFunction` | Get the algebraic expression that gives the interaction energy between two particles |
    | `setEnergyFunction` | Set the algebraic expression that gives the interaction energy between two particles |
    | `getNonbondedMethod` | Get the method used for handling long range nonbonded interactions. |
    | `setNonbondedMethod` | Set the method used for handling long range nonbonded interactions. |
    | `getCutoffDistance` | Get the cutoff distance (in nm) being used for nonbonded interactions. |
    | `setCutoffDistance` | Set the cutoff distance (in nm) being used for nonbonded interactions. |
    | `getUseSwitchingFunction` | Get whether a switching function is applied to the interaction. |
    | `setUseSwitchingFunction` | Set whether a switching function is applied to the interaction. |
    | `getSwitchingDistance` | Get the distance at which the switching function begins to reduce the interaction. |
    | `setSwitchingDistance` | Set the distance at which the switching function begins to reduce the interaction. |
    | `getUseLongRangeCorrection` | Get whether to add a correction to the energy to compensate for the cutoff and switching function. |
    | `setUseLongRangeCorrection` | Set whether to add a correction to the energy to compensate for the cutoff and switching function. |
    | `addPerParticleParameter` | Add a new per-particle parameter that the interaction may depend on. |
    | `getPerParticleParameterName` | Get the name of a per-particle parameter. |
    | `setPerParticleParameterName` | Set the name of a per-particle parameter. |
    | `addGlobalParameter` | Add a new global parameter that the interaction may depend on. |
    | `getGlobalParameterName` | Get the name of a global parameter. |
    | `setGlobalParameterName` | Set the name of a global parameter. |
    | `getGlobalParameterDefaultValue` | Get the default value of a global parameter. |
    | `setGlobalParameterDefaultValue` | Set the default value of a global parameter. |
    | `addParticle` | Add the nonbonded force parameters for a particle. |
    | `getParticleParameters` | Get the nonbonded force parameters for a particle. |
    | `setParticleParameters` | Set the nonbonded force parameters for a particle. |
    | `addExclusion` | Add a particle pair to the list of interactions that should be excluded. |
    | `getExclusionParticles` | Get the particles in a pair whose interaction should be excluded. |
    | `setExclusionParticles` | Set the particles in a pair whose interaction should be excluded. |
    | `createExclusionsFromBonds` | Identify exclusions based on the molecular topology. |
    | `addTabulatedFunction` | Add a tabulated function that may appear in the energy expression. |
    | `getTabulatedFunction` | Get a const reference to a tabulated function that may appear in the energy expression. |
    | `getTabulatedFunction` | Get a reference to a tabulated function that may appear in the energy expression. |
    | `getTabulatedFunctionName` | Get the name of a tabulated function that may appear in the energy expression. |
    | `addFunction` | Add a tabulated function that may appear in the energy expression. |
    | `getFunctionParameters` | Get the parameters for a tabulated function that may appear in the energy expression. |
    | `setFunctionParameters` | Set the parameters for a tabulated function that may appear in the energy expression. |
    | `addInteractionGroup` | Add an interaction group. |
    | `getInteractionGroupParameters` | Get the parameters for an interaction group. |
    | `setInteractionGroupParameters` | Set the parameters for an interaction group. |
    | `updateParametersInContext` | Update the per-particle parameters in a `Context` to match those stored in this `Force` object. |
    | `usesPeriodicBoundaryConditions` | Returns whether or not this force makes use of periodic boundary conditions. |

    Enum: NonbondedMethod

    |  |  |
    | --- | --- |
    | **NoCutoff** | No cutoff is applied to nonbonded interactions. The full set of N^2 interactions is computed exactly. This necessarily means that periodic boundary conditions cannot be used. This is the default. |
    | **CutoffNonPeriodic** | Interactions beyond the cutoff distance are ignored. |
    | **CutoffPeriodic** | Periodic boundary conditions are used, so that each particle interacts only with the nearest periodic copy of each other particle. Interactions beyond the cutoff distance are ignored. |

    `CustomNonbondedForce`(*const* std::string &*energy*)¶
    :   Create a `CustomNonbondedForce`.

        |  |  |
        | --- | --- |
        | Parameters: |  |

        - **energy** – an algebraic expression giving the interaction energy between two particles as a function of r, the distance between them, as well as any global and per-particle parameters

    `CustomNonbondedForce`(*const* CustomNonbondedForce &*rhs*)¶

    `~CustomNonbondedForce`()¶

    int `getNumParticles`() *const*¶
    :   Get the number of particles for which force field parameters have been defined.

    int `getNumExclusions`() *const*¶
    :   Get the number of particle pairs whose interactions should be excluded.

    int `getNumPerParticleParameters`() *const*¶
    :   Get the number of per-particle parameters that the interaction depends on.

    int `getNumGlobalParameters`() *const*¶
    :   Get the number of global parameters that the interaction depends on.

    int `getNumTabulatedFunctions`() *const*¶
    :   Get the number of tabulated functions that have been defined.

    int `getNumFunctions`() *const*¶
    :   Get the number of tabulated functions that have been defined.

        Deprecated

        This method exists only for backward compatibility. Use `getNumTabulatedFunctions()` instead.

    int `getNumInteractionGroups`() *const*¶
    :   Get the number of interaction groups that have been defined.

    *const* std::string &`getEnergyFunction`() *const*¶
    :   Get the algebraic expression that gives the interaction energy between two particles

    void `setEnergyFunction`(*const* std::string &*energy*)¶
    :   Set the algebraic expression that gives the interaction energy between two particles

    NonbondedMethod `getNonbondedMethod`() *const*¶
    :   Get the method used for handling long range nonbonded interactions.

    void `setNonbondedMethod`(NonbondedMethod *method*)¶
    :   Set the method used for handling long range nonbonded interactions.

    double `getCutoffDistance`() *const*¶
    :   Get the cutoff distance (in nm) being used for nonbonded interactions. If the NonbondedMethod in use is NoCutoff, this value will have no effect.

        |  |  |
        | --- | --- |
        | Returns: | the cutoff distance, measured in nm |

    void `setCutoffDistance`(double *distance*)¶
    :   Set the cutoff distance (in nm) being used for nonbonded interactions. If the NonbondedMethod in use is NoCutoff, this value will have no effect.

        |  |  |
        | --- | --- |
        | Parameters: |  |

        - **distance** – the cutoff distance, measured in nm

    bool `getUseSwitchingFunction`() *const*¶
    :   Get whether a switching function is applied to the interaction. If the nonbonded method is set to NoCutoff, this option is ignored.

    void `setUseSwitchingFunction`(bool *use*)¶
    :   Set whether a switching function is applied to the interaction. If the nonbonded method is set to NoCutoff, this option is ignored.

    double `getSwitchingDistance`() *const*¶
    :   Get the distance at which the switching function begins to reduce the interaction. This must be less than the cutoff distance.

    void `setSwitchingDistance`(double *distance*)¶
    :   Set the distance at which the switching function begins to reduce the interaction. This must be less than the cutoff distance.

    bool `getUseLongRangeCorrection`() *const*¶
    :   Get whether to add a correction to the energy to compensate for the cutoff and switching function. This has no effect if periodic boundary conditions are not used.

    void `setUseLongRangeCorrection`(bool *use*)¶
    :   Set whether to add a correction to the energy to compensate for the cutoff and switching function. This has no effect if periodic boundary conditions are not used.

    int `addPerParticleParameter`(*const* std::string &*name*)¶
    :   Add a new per-particle parameter that the interaction may depend on.

        |  |  |
        | --- | --- |
        | Parameters: |  |

        - **name** – the name of the parameter

        |  |  |
        | --- | --- |
        | Returns: | the index of the parameter that was added |

    *const* std::string &`getPerParticleParameterName`(int *index*) *const*¶
    :   Get the name of a per-particle parameter.

        |  |  |
        | --- | --- |
        | Parameters: |  |

        - **index** – the index of the parameter for which to get the name

        |  |  |
        | --- | --- |
        | Returns: | the parameter name |

    void `setPerParticleParameterName`(int *index*, *const* std::string &*name*)¶
    :   Set the name of a per-particle parameter.

        |  |  |
        | --- | --- |
        | Parameters: |  |

        - **index** – the index of the parameter for which to set the name
        - **name** – the name of the parameter

    int `addGlobalParameter`(*const* std::string &*name*, double *defaultValue*)¶
    :   Add a new global parameter that the interaction may depend on.

        |  |  |
        | --- | --- |
        | Parameters: |  |

        - **name** – the name of the parameter
        - **defaultValue** – the default value of the parameter

        |  |  |
        | --- | --- |
        | Returns: | the index of the parameter that was added |

    *const* std::string &`getGlobalParameterName`(int *index*) *const*¶
    :   Get the name of a global parameter.

        |  |  |
        | --- | --- |
        | Parameters: |  |

        - **index** – the index of the parameter for which to get the name

        |  |  |
        | --- | --- |
        | Returns: | the parameter name |

    void `setGlobalParameterName`(int *index*, *const* std::string &*name*)¶
    :   Set the name of a global parameter.

        |  |  |
        | --- | --- |
        | Parameters: |  |

        - **index** – the index of the parameter for which to set the name
        - **name** – the name of the parameter

    double `getGlobalParameterDefaultValue`(int *index*) *const*¶
    :   Get the default value of a global parameter.

        |  |  |
        | --- | --- |
        | Parameters: |  |

        - **index** – the index of the parameter for which to get the default value

        |  |  |
        | --- | --- |
        | Returns: | the parameter default value |

    void `setGlobalParameterDefaultValue`(int *index*, double *defaultValue*)¶
    :   Set the default value of a global parameter.

        |  |  |
        | --- | --- |
        | Parameters: |  |

        - **index** – the index of the parameter for which to set the default value
        - **defaultValue** – the default value of the parameter

    int `addParticle`(*const* std::vector<double> &*parameters* = std::vector< double >())¶
    :   Add the nonbonded force parameters for a particle. This should be called once for each particle in the `System`. When it is called for the i’th time, it specifies the parameters for the i’th particle.

        |  |  |
        | --- | --- |
        | Parameters: |  |

        - **parameters** – the list of parameters for the new particle

        |  |  |
        | --- | --- |
        | Returns: | the index of the particle that was added |

    void `getParticleParameters`(int *index*, std::vector<double> &*parameters*) *const*¶
    :   Get the nonbonded force parameters for a particle.

        |  |  |
        | --- | --- |
        | Parameters: |  |

        - **index** – the index of the particle for which to get parameters
        - **parameters** – [out] the list of parameters for the specified particle

    void `setParticleParameters`(int *index*, *const* std::vector<double> &*parameters*)¶
    :   Set the nonbonded force parameters for a particle.

        |  |  |
        | --- | --- |
        | Parameters: |  |

        - **index** – the index of the particle for which to set parameters
        - **parameters** – the list of parameters for the specified particle

    int `addExclusion`(int *particle1*, int *particle2*)¶
    :   Add a particle pair to the list of interactions that should be excluded.

        In many cases, you can use `createExclusionsFromBonds()` rather than adding each exclusion explicitly.

        |  |  |
        | --- | --- |
        | Parameters: |  |

        - **particle1** – the index of the first particle in the pair
        - **particle2** – the index of the second particle in the pair

        |  |  |
        | --- | --- |
        | Returns: | the index of the exclusion that was added |

    void `getExclusionParticles`(int *index*, int &*particle1*, int &*particle2*) *const*¶
    :   Get the particles in a pair whose interaction should be excluded.

        |  |  |
        | --- | --- |
        | Parameters: |  |

        - **index** – the index of the exclusion for which to get particle indices
        - **particle1** – [out] the index of the first particle in the pair
        - **particle2** – [out] the index of the second particle in the pair

    void `setExclusionParticles`(int *index*, int *particle1*, int *particle2*)¶
    :   Set the particles in a pair whose interaction should be excluded.

        |  |  |
        | --- | --- |
        | Parameters: |  |

        - **index** – the index of the exclusion for which to set particle indices
        - **particle1** – the index of the first particle in the pair
        - **particle2** – the index of the second particle in the pair

    void `createExclusionsFromBonds`(*const* std::vector<std::pair<int, int>> &*bonds*, int *bondCutoff*)¶
    :   Identify exclusions based on the molecular topology. Particles which are separated by up to a specified number of bonds are added as exclusions.

        |  |  |
        | --- | --- |
        | Parameters: |  |

        - **bonds** – the set of bonds based on which to construct exclusions. Each element specifies the indices of two particles that are bonded to each other.
        - **bondCutoff** – pairs of particles that are separated by this many bonds or fewer are added to the list of exclusions

    int `addTabulatedFunction`(*const* std::string &*name*, TabulatedFunction \**function*)¶
    :   Add a tabulated function that may appear in the energy expression.

        |  |  |
        | --- | --- |
        | Parameters: |  |

        - **name** – the name of the function as it appears in expressions
        - **function** – a `TabulatedFunction` object defining the function. The `TabulatedFunction` should have been created on the heap with the “new” operator. The `Force` takes over ownership of it, and deletes it when the `Force` itself is deleted.

        |  |  |
        | --- | --- |
        | Returns: | the index of the function that was added |

    *const* TabulatedFunction &`getTabulatedFunction`(int *index*) *const*¶
    :   Get a const reference to a tabulated function that may appear in the energy expression.

        |  |  |
        | --- | --- |
        | Parameters: |  |

        - **index** – the index of the function to get

        |  |  |
        | --- | --- |
        | Returns: | the `TabulatedFunction` object defining the function |

    TabulatedFunction &`getTabulatedFunction`(int *index*)¶
    :   Get a reference to a tabulated function that may appear in the energy expression.

        |  |  |
        | --- | --- |
        | Parameters: |  |

        - **index** – the index of the function to get

        |  |  |
        | --- | --- |
        | Returns: | the `TabulatedFunction` object defining the function |

    *const* std::string &`getTabulatedFunctionName`(int *index*) *const*¶
    :   Get the name of a tabulated function that may appear in the energy expression.

        |  |  |
        | --- | --- |
        | Parameters: |  |

        - **index** – the index of the function to get

        |  |  |
        | --- | --- |
        | Returns: | the name of the function as it appears in expressions |

    int `addFunction`(*const* std::string &*name*, *const* std::vector<double> &*values*, double *min*, double *max*)¶
    :   Add a tabulated function that may appear in the energy expression.

        Deprecated

        This method exists only for backward compatibility. Use `addTabulatedFunction()` instead.

    void `getFunctionParameters`(int *index*, std::string &*name*, std::vector<double> &*values*, double &*min*, double &*max*) *const*¶
    :   Get the parameters for a tabulated function that may appear in the energy expression.

        Deprecated

        This method exists only for backward compatibility. Use getTabulatedFunctionParameters() instead. If the specified function is not a `Continuous1DFunction`, this throws an exception.

    void `setFunctionParameters`(int *index*, *const* std::string &*name*, *const* std::vector<double> &*values*, double *min*, double *max*)¶
    :   Set the parameters for a tabulated function that may appear in the energy expression.

        Deprecated

        This method exists only for backward compatibility. Use setTabulatedFunctionParameters() instead. If the specified function is not a `Continuous1DFunction`, this throws an exception.

    int `addInteractionGroup`(*const* std::set<int> &*set1*, *const* std::set<int> &*set2*)¶
    :   Add an interaction group. An interaction will be computed between every particle in set1 and every particle in set2.

        |  |  |
        | --- | --- |
        | Parameters: |  |

        - **set1** – the first set of particles forming the interaction group
        - **set2** – the second set of particles forming the interaction group

        |  |  |
        | --- | --- |
        | Returns: | the index of the interaction group that was added |

    void `getInteractionGroupParameters`(int *index*, std::set<int> &*set1*, std::set<int> &*set2*) *const*¶
    :   Get the parameters for an interaction group.

        |  |  |
        | --- | --- |
        | Parameters: |  |

        - **index** – the index of the interaction group for which to get parameters
        - **set1** – [out] the first set of particles forming the interaction group
        - **set2** – [out] the second set of particles forming the interaction group

    void `setInteractionGroupParameters`(int *index*, *const* std::set<int> &*set1*, *const* std::set<int> &*set2*)¶
    :   Set the parameters for an interaction group.

        |  |  |
        | --- | --- |
        | Parameters: |  |

        - **index** – the index of the interaction group for which to set parameters
        - **set1** – the first set of particles forming the interaction group
        - **set2** – the second set of particles forming the interaction group

    void `updateParametersInContext`(Context &*context*)¶
    :   Update the per-particle parameters in a `Context` to match those stored in this `Force` object. This method provides an efficient method to update certain parameters in an existing `Context` without needing to reinitialize it. Simply call `setParticleParameters()` to modify this object’s parameters, then call `updateParametersInContext()` to copy them over to the `Context`.

        This method has several limitations. The only information it updates is the values of per-particle parameters. All other aspects of the `Force` (the energy function, nonbonded method, cutoff distance, etc.) are unaffected and can only be changed by reinitializing the `Context`. Also, this method cannot be used to add new particles, only to change the parameters of existing ones.

    bool `usesPeriodicBoundaryConditions`() *const*¶
    :   Returns whether or not this force makes use of periodic boundary conditions.

        |  |  |
        | --- | --- |
        | Returns: | true if force uses PBC and false otherwise |

High performance molecular simulation on GPUs


### Navigation

- Core Objects
- Forces
- Integrators
- Extras

©2015, Stanford University and the Authors.
|
Powered by Sphinx 1.3.1
& Alabaster 0.7.7
|
Page source
